# A bolt from the blue in a testicular mass - continuous Splenogonadal fusion with maturation arrest

**DOI:** 10.4322/acr.2023.442

**Published:** 2023-08-07

**Authors:** Thotadamane Nagaraja Chandrashekhar, Priyadharshini Bargunam, Ragashree Apparasanahalli Siddalingamurthy

**Affiliations:** 1 Shimoga Institute of Medical Sciences, Department of Pathology, Shivamogga, Karnataka, India; 2 Vardhaman Mahavir Medical College and Safdarjung Hospital, Department of Pathology, New Delhi, India

**Keywords:** Splenogonadal fusion, Continuous variant, testicular mass, orchiectomy, maturation arrest

## Abstract

Splenogonadal fusion is an infrequent cause of testicular or scrotal swelling with less than 250 cases reported. We report the case of a 27-year-old male who presented with painless scrotal swelling. The sonography showed a homogeneous, well-encapsulated left extratesticular mass, which was surgically removed. The gross examination revealed a grey-brown tissue below the left testis. The microscopy of the grey-brown mass revealed splenic tissue, and the testis showed maturation arrest, resulting in the diagnosis of splenogonadal fusion. These can be easily mistaken for a tumor, especially in this age group. Reporting such an entity increases awareness among clinicians, radiologists and pathologists, which will aid in preventing an orchiectomy for these patients.

## INTRODUCTION

Splenogonadal fusion (SGF) is a rare congenital anomaly that results from an abnormal fusion between the spleen and the gonads between the 5-8 weeks of gestation when the splenic anlage is close to the left urogenital ridge.^1^ Since the first description in 1883 by Bostroem,^2^ less than 250 cases have been reported in the literature. SGF usually presents as a scrotal or abdominal mass and is usually benign;^3^ however, it is often confused with testicular tumors. SGF may be associated with testicular tumors when associated with cryptorchidism.^4^ Putschar and Manion^5^ in 1956 classified splenogonodal fusion as the continuous and discontinuous types based on a connection between the testis and spleen. The continuous type is relatively common and is associated with several congenital anomalies like micrognathia, macroglossia, anal atresia, and pulmonary hypoplasia.^6^ Preoperative diagnosis is challenging due to its rarity, lack of distinct imaging findings,^7^ and restriction of testicular FNACs and biopsy with the fear of tumor seeding and hence mandates an orchiectomy in most cases.

## CASE REPORT

A 27-year-old man was referred for evaluation of a palpable, mobile, and painless left scrotal mass. The patient did not report any history of trauma or cryptorchidism. No external anomalies/malformations were noted. The physical examination lacked scrotal inflammatory signs. The mass was located inside the scrotum beneath the epididymis attached to the testis and was slightly painful on palpation. The border between the solid mass, testis, and epididymis was vague. The laboratory results were unremarkable. Serum alpha-fetoprotein, beta HCG, and lactate dehydrogenase levels were normal. The sonography showed a homogeneous, well-encapsulated left extra testicular mass with a cord showing the same echogenicity as that of the testis. The patient underwent exploratory left inguinal orchiectomy, and the mass was excised along with the spermatic cord.

**Figure 1:**
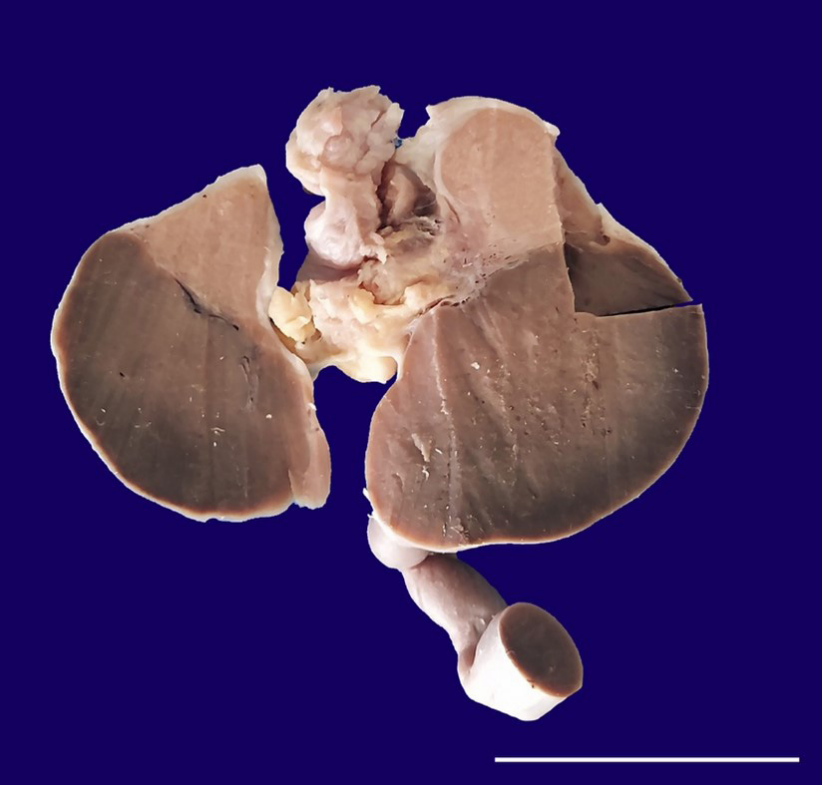
Cross-section of the specimen showing the testis attached to ectopic splenic tissue (grey-brown mass) with a connecting cord.

The specimen was preserved in 10% formalin and was sent to the department of pathology post-fixation. Grossly, the specimen showed a grey-brown mass attached to the inferior pole of the testis (Figure 1).

The length of the cord and the testicular measurements were normal. The cut surface showed the inferior aspect of the testis and a part of the epididymis attached to the grey-brown mass (Figure 1). The epididymis and the spermatic cord were grossly unremarkable. The tunica was intact, and the string sign was positive on the testicular side. However, the grey-brown homogenous mass showed a negative string sign. There were no areas of necrosis, hemorrhage, or capsular breech. Sections were taken from the testis, the grey-brown mass, the interface of both, and sections from the cord and epididymis.

**Figure 2:**
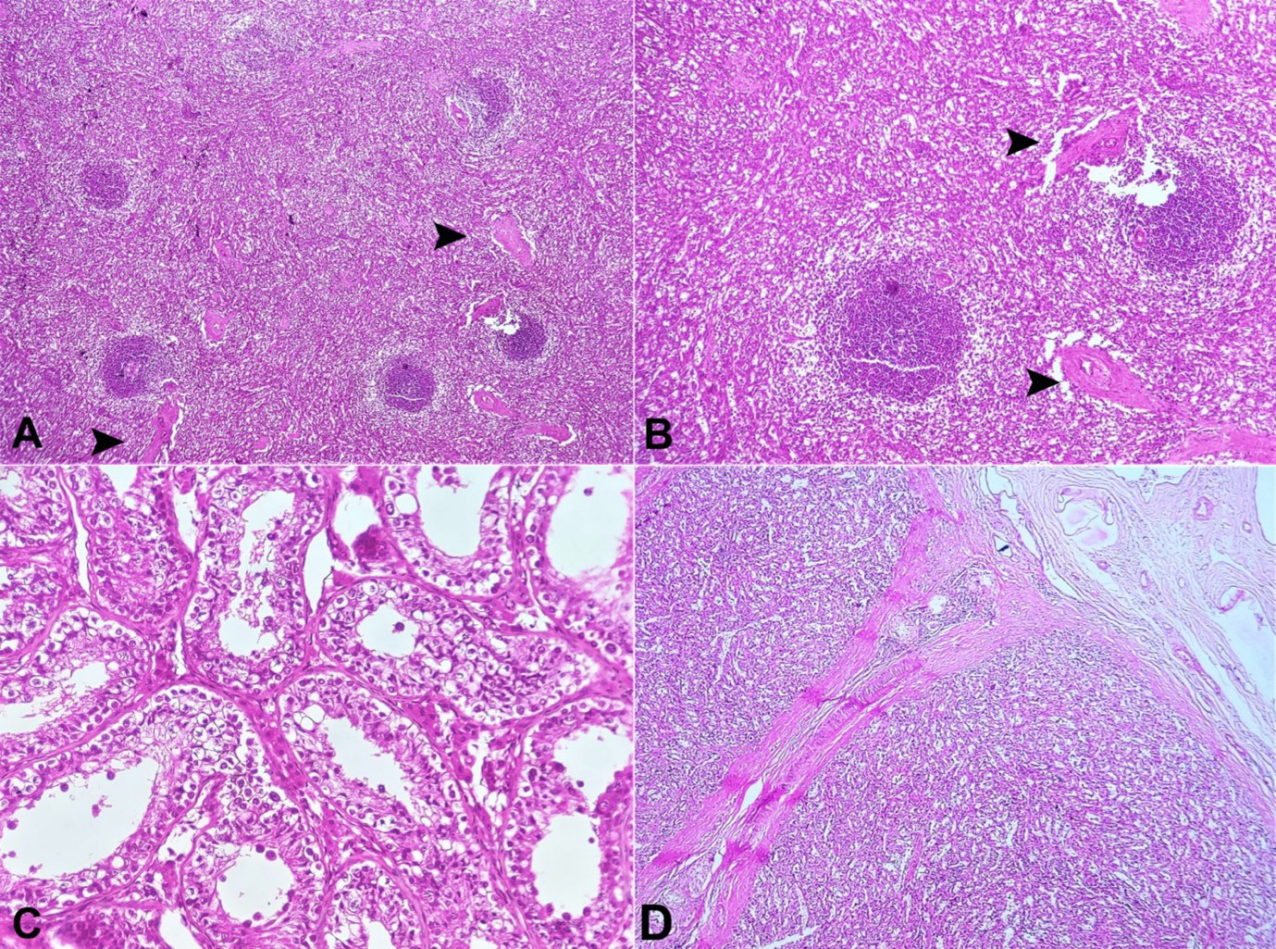
**A & B -** Low and high magnification images of the ectopic splenic tissue showing white pulp, red pulp, and intertwining trabeculae (arrowheads) (H&E, 10x and 40x respectively); **C** - High magnification image of seminiferous tubules showing numerous spermatogonia, few spermatocytes and absent mature sperms suggestive of maturation arrest (H&E, 40x); **D** - Section from the cord showing splenic tissue separated by fibrous septae (H&E, 20 x).

Paraffin-embedded H&E-stained sections from the mass showed a well-circumscribed lymphoid tissue covered with a thick fibrous capsule organized in follicles and separated by sinuses to form red and white pulp with intertwining fibrous septae. (Figures 2A and 2B). Section from the testicular tissue showed seminiferous tubules showing numerous spermatogonia, few spermatocytes and absent mature sperms suggestive of maturation arrest. (Figure 2C). Sections from the cord showed the entire cord replaced by the splenic tissue confirming the splenogonadal fusion (continuous variant) (Figure 2D).

**Figure 3:**
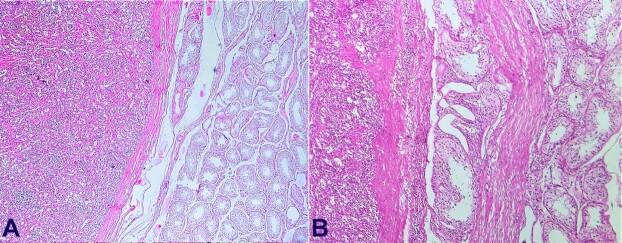
**A and B -** Low power and high power images of the junction of the testicular and splenic tissue separated by a fibrous capsule (H&E, 10 x and 20x, respectively).

Figures 3A and 3B show seminiferous tubules adjacent to splenic tissue separated by a capsule.

Owing to the histological diagnosis, malignancy was ruled out. However, the patient is still in a half-yearly follow-up. The patient is doing well without any complaints.

## DISCUSSION

Though first reported by Bostroem^2^ in 1883, the first detailed review of 30 SGF cases was published by Putschar and Manion^5^ in 1956, wherein an SGF classification system was established. SGF can be continuous (55%), wherein there is an anatomical continuation between the spleen and the gonad, or discontinuous type (45%), wherein an anatomical continuity is lacking. Though there is no prognostic difference between the two groups, the continuous type is associated with congenital anomalies. Cryptorchidism and inguinal hernias are the most commonly associated anomalies.^6^ The other anomalies reported are cardiac defects, limb bud abnormalities, micrognathia, hypoglossia, craniosynostosis, and spina bifida. In our case, the patient had no associated congenital anomalies or cryptorchidism; however, the testicular tissue showed maturation arrest. No reports of maturation arrest associated with splenogonadal fusion were found. Splenogonadal fusion limb defect syndrome (SGFLD), characterized by SGF with skeletal and limb defects, has perinatal mortality of 40-50%.^7^ In 1990, Carragher^8^ reviewed 123 SGF cases, with 70% of pediatric cases. Subsequently, Malik and Liu^9^ in 2013 published a review of 61 additional SGF cases. Chen et al.^10^ reviewed another 40 cases in 2021. Table 1 shows the comparative analysis of the epidemiological factors in the major reviews of splenogonadal fusion.

**Table 1:** The Comparison of the conclusions of the three major reviews of SGF with the reported case

	Carragher^8^	Malik and Liu^9^	Chen et al.^10^
Years Covered	1890- 1990	1890- 2013	2014- 2021
Number of cases	123	123+ 61	41
< 20 years	88 (71.5%)	125 (68%)	26 (63.4%)
>20 years	35 (28.5%)	51 (27%)	15 (36.5%)
Male: Female Ratio	16.6: 1	14.3:1	40:1
Continuous Type	69 (56%)	105 (58%)	19 (46.34%)
Discontinuous Type	54 (44%)	76 (42%)	22 (53.65%)
Associated Anomalies	24 (19.5%)	47 (26%)	6 (14.6%)
Mode of Presentation			
Autopsy	22 (18%)	33 (18%)	Nil
Scrotal swelling	51 (41.5%)	78 (42%)	28 (68.29%)
Left inguinal Hernia	23 (18.7%)	26 (14%)	5 (12.20%)
Left Undescended testis	18 (14.6%)	31 (17%)	11 (26.83%)
Acute Abdomen	5 (4%)	5 (3%)	Nil
Torsion	4 (3.3%)	5 (3%)	Nil
Others	0	6 (4%)	3 (7.3%)
Unnecessary Orchiectomy	45 (37%)	15/61 (24%)	14 (34.15%)

SGF is seen predominantly in males with a male: female ratio of 15:1. This is due to the superficial location of the male gonads compared to females. One case is reported in a genotypic XY but phenotypic female.^11^ SGF is predominantly seen on the left side. SGF is more commonly reported in children and adolescents. More than 50% of the case reports are under 10 years of age, and 70% are under 20.^6^

The clinical presentation is non-specific and cannot point toward a definitive preoperative diagnosis. They usually present as a testicular or abdominal mass and may be an incidental finding as most of them are asymptomatic. They can rarely present as an acute emergency with torsion, rupture, or malignant transformation.^4^ Few cases are diagnosed post-mortem during an autopsy.^9^

In cases of continuous SGF in a middle-aged man with complete descent of the testis, as in our case, it is almost impossible to come to a preoperative clinical diagnosis as it can easily be confused with torsion, tumors, and infections. Though there were 1 or 2 instances where a preoperative diagnosis of SGF was made based on imaging, resulting in testis preserving surgeries, in most of cases, SGF patients undergo an orchiectomy as they mimic a testicular tumor.^12^ Torsion presents with acute pain and can be ruled out with imaging in most cases, and tumors usually have vascular changes even in Ultrasonogram and are accompanied by biochemical and tumor markers. These subtle changes, with a high index of suspicion can help to come to a preoperative diagnosis. Due to the fear of tumor seeding, testicular FNACs are also generally avoided. Besides, the incidence of SGF is also too low to consider it as a differential diagnosis in every scrotal swelling.

The testis can be spared by detailed radiological evaluation pre-operatively using B-type Ultrasonography (USG), Computed Tomography (CT), and Magnetic Resonance Imaging (MRI) and a high index of suspicion by the treating physician. Clinicians and radiologists can make it a norm to always comment if the spleen is in the left hypochondrium; in any scrotal swelling evaluation In children aged between one and five years, the ultrasonography reveals a low reflectivity micronodular pattern in both SGF and the normally located spleen due to the development of the lymphoid system and white pulp follicles.^13^ Malignant lesions show more disorganized vascularity and would be expected to show washout of ultrasound contrast compared to SGF. Doppler visualizes a vascular mass in the testis's upper pole in SGF along with a normally located spleen and can help confirm the diagnosis of SGF. Imaging through 99m Tc-sulfur colloid liver spleen scan detects accessory spleen, which is even more reliable for SGF diagnosis.^14^ On table FNAC and Frozen section can also help in preoperative diagnosis. Laparoscopy is a very reliable diagnostic and therapeutic method; however, the discontinuous types can still cause doubts about a malignant tumor without a frozen section.

SGF, which presents as cryptorchidism, requires separate two-stage laparoscopic staged Fowler-Stephen orchiopexy.^10^ In the absence of an associated malignancy, complete excision of the spleen is all that is needed; however, unnecessary orchiectomies are reported. Carragher^8^ reported that 37% of the 123 cases had an unnecessary orchiectomy, whereas Chen et al.^10^ 31 years later, still reported 34% unnecessary orchiectomy in their review. Kadouri et al.^15^ proposed a diagnostic algorithm for chronic testicular nodule (partially followed in the index case) with the help of tumor markers and scrotal ultrasound. They propose that in cases of high tumor markers an orchiectomy should be performed; in cases with normal markers, and inconclusive ultrasonographic examination, a Tc 99 Scintigraphy can be done; and in cases with normal markers and suspicious sonogram, surgical exploration should be done. A missense or a frame-shift mutation in the gene encoding SET binding protein 1 (SETBP1) (c.2608G>A [p.Gly870Ser]), and a mutation in the oligophrenin-1 gene (OPHN1) are identified in these patients and could be a vital pathways to be targeted for therapy in the future.^10^

## CONCLUSION

SGF is an important differential diagnosis to be considered in an indolent testicular or scrotal mass; as they are usually benign, a preoperative diagnosis can help the surgeon to preserve the testis in these young males.
